# Case Report: Radiological–Pathological Correlation of Ectopic Pancreatic Tissue as Anterior Mediastinal Mass

**DOI:** 10.1155/crra/4836145

**Published:** 2026-09-29

**Authors:** Zehao Quan, David Luong, John Coucher

**Affiliations:** ^1^ Faculty of Medicine, University of Queensland, Herston, Queensland, Australia, uq.edu.au; ^2^ Department of Diagnostic Radiology and Medical Imaging, Princess Alexandra Hospital, Woolloongabba, Queensland, Australia, qld.gov.au

**Keywords:** anterior mediastinal mass, case report, ectopic pancreas, heterotopic pancreas, radiological–pathological correlation

## Abstract

Ectopic pancreatic tissue presenting as large anterior mediastinal mass is exceedingly rare and poses a significant diagnostic challenge, which could mimic common malignancies such as thymoma, teratoma or lymphoma. We report a case of a 49‐year‐old female who presented with pleuritic chest pain, new‐onset atrial fibrillation with rapid ventricular response and nonsustained ventricular tachycardia. Computer tomography pulmonary angiogram (CTPA) identified a large peripherally enhancing anterior mediastinal mass with extensive central necrosis. The lesion exerted a significant mass effect, compressing the pulmonary trunk and displacing the ascending aorta. Initial CT‐guided core biopsy was nondiagnostic, yielding only necrotic debris and neutrophils. Given the diagnostic uncertainty and the patient′s worsening symptoms, a surgical biopsy via a Chamberlain procedure was performed, revealing thick, black necrotic fluid with histology confirming ectopic pancreatic tissue. This case demonstrates ectopic pancreatic tissue as a rare differential of a necrotic anterior mediastinal mass. Early recognition is vital to guide appropriate surgical management and prevent morbidity related to pancreatic enzymatic action on surrounding key anatomical structures and the misdiagnosis of more common mediastinal neoplasms.

## 1. Introduction

Ectopic pancreas is defined as the presence of pancreatic tissue in extra pancreatic locations, lacking anatomical or vascular continuity with the main pancreatic gland [1, 2]. Ectopic pancreas is a congenital anomaly caused by the abnormal separation of embryonic pancreatic tissue during foetal development [3]. As the dorsal and ventral pancreatic buds rotate and fuse [4], small fragments of pancreatic tissue may be misplaced along the gastrointestinal tract [3], most commonly in the stomach [5, 6], duodenum [5, 7, 8] and jejunum [9, 10]. Thoracic involvement is exceedingly rare [11]. Clinically, ectopic pancreatic tissue can mimic more common anterior mediastinal neoplasms, such as thymoma, germ cell tumours or lymphoma, particularly when they present with non‐specific systemic symptoms or mass effect on adjacent cardiothoracic structures [12, 13]. Consequently, achieving a preoperative diagnosis remains a significant challenge, as standard percutaneous biopsies may only yield necrotic or nondiagnostic material due to the cystic and enzymatic nature of the tissue. This case report describes a unique presentation of a massive, necrotic anterior mediastinal ectopic pancreas in a 49‐year‐old female, highlighting the critical role of radiological‐pathological correlation in reaching a definitive diagnosis for this rare differential. This case report was prepared and is reported in accordance with the CARE (CAse REport) guideline [14], and a completed CARE checklist is provided as Supporting Information 2.

## 2. Case Presentation

The chronology of this episode of care is summarised in Table 1.

**Table 1:** Structured timeline of the episode of care. Dates are given as month and year, or as days relative to the index admission, to preserve patient confidentiality.

Time point	Event	Key findings
Oct 2025	Symptom onset	Two‐month history of intermittent dizziness (mixed presyncope and swaying, worse with head movement, not clearly postural or exertional), dyspnoea and global headaches. Nocturnal palpitations and nightmares waking her from sleep.
Dec 2025	First ED presentation	Physical examination, including full neurological and cranial nerve assessment, entirely normal. Chest radiograph reported as normal cardiomediastinal contour. Discharged for outpatient echocardiogram and Holter monitor via GP.
Dec 2025–Feb 2026	Interval	Symptoms persisted intermittently; outpatient investigations arranged in the community.
Feb 2026, Day 0 (01:00)	Acute representation	Woke with sudden‐onset palpitations, dizziness and generalised chest tightness, 5/10, nonradiating. AF with rapid ventricular response ~200 bpm; three runs of NSVT, longest 10 s. Temperature 38°C, heart rate 182 bpm, respiratory rate 28. Intravenous fentanyl required.
Day 0	Laboratory investigations	WCC 23.2 × 10^9^/L; CRP 239 mg/L; ESR 84 mm/h; troponin I 57 ng/L; D‐dimer 1.33 mg/L.
Day 0	CTPA	Negative for pulmonary embolism. Well‐circumscribed anterior mediastinal mass 65 × 76 × 91 mm; peripheral enhancement 42 HU, central hypoattenuation 18 HU; ascending aorta displaced posteriorly; RV dilatation with interventricular septal flattening; 7 mm anterior pericardial effusion; prominent left supraclavicular nodes to 5 mm.
Day 1	Laboratory investigations	WCC 26.3 × 10^9^/L with neutrophilia 24.36 × 10^9^/L. Troponin I peak 156 ng/L during tachycardia. Blood cultures and respiratory viral panel negative. Peripheral flow cytometry: small monoclonal CD8+ T‐cell population (0.4% of total cells).
Day 1	TTE	Extrinsic compression of the RVOT; 7 mm anterior pericardial effusion.
Day 1	CT‐guided core biopsy	Six 18‐gauge cores. Predominantly necrotic, “abscess‐like” tissue with neutrophils, macrophages and granulation tissue; sclerotic, compartmentalised background stroma. Nondiagnostic.
Day 3	18F‐FDG PET‐CT torso	Moderately intense peripheral FDG avidity, SUVmax 7.27; photopenic central component. No FDG‐avid lymphadenopathy or distant disease above or below the diaphragm.
Day 5	Chamberlain procedure (left anterior mediastinotomy)	Thick‐walled, fluid‐filled lesion containing thick black necrotic fluid. Histology: densely fibrotic tissue with mixed chronic inflammation and ectopic pancreatic tissue comprising acini, ducts and islets in normal lobular architecture. No teratomatous elements, no lymphoproliferative disorder, no neoplasia. Diagnosis: Heinrich Type I heterotopic pancreas.
Day 7 post‐Chamberlain	Wound complication	Erythema, pain and persistent ooze at the mini‐thoracotomy site, consistent with fat necrosis from enzymatic action rather than primary infection. Urgent irrigation and debridement revealed a tract through the deeper suture layers into the mediastinum. VAC dressing applied.
Early Apr 2026	Definitive resection	Median sternotomy and excision of the mediastinal mass, densely adherent to the left upper lobe at the previous biopsy site, requiring partial lung resection. Left phrenic nerve, brachiocephalic vein and pericardium preserved.
Day 5 post‐sternotomy	Postoperative recovery	Wounds healing well; mobilising independently. Discharged with plan for community suture removal. Arrhythmias resolved.
6 weeks postresection	Outpatient review	Reviewed in the cardiothoracic clinic. Complete resolution of all presenting symptoms, with no recurrence of arrhythmia.

Abbreviations: AF, atrial fibrillation; CRP, C‐reactive protein; CTPA, computed tomography pulmonary angiogram; ESR, erythrocyte sedimentation rate; NSVT, nonsustained ventricular tachycardia; RV, right ventricle; RVOT, right ventricular outflow tract; TTE, transthoracic echocardiogram; VAC, vacuum‐assisted closure; WCC, white cell count.

### 2.1. Initial Presentation

In late December 2025, the 49‐year‐old female patient presented to ED with a 2‐month history of intermittent dizziness, dyspnoea and global headaches. She described the dizziness as mixed presyncope and swaying sensation that worsened with head movement but was not clearly postural or exertional. Notably, she reported nocturnal palpitations and nightmares that woke her from sleep. Physical examination, including a comprehensive neurological and cranial nerve assessment, was entirely normal. A chest x‐ray performed during this visit was reported as showing normal cardiomediastinal contour. She was discharged with advice to undergo an outpatient echocardiogram and Holter monitor with GP.

### 2.2. Acute Representation

The patient represented 2 months later to ED after waking at 1 am with sudden‐onset palpitations, dizziness and generalised chest tightness. The pain was described as 5/10 in intensity and nonradiating. The patient was in atrial fibrillation with a rapid ventricular response of approximately 200 bpm. Telemetry also recorded three separate runs of nonsustained ventricular tachycardia, the longest lasting 10 s. She was febrile with a temperature of 38 °C, tachycardic with a heart rate of 182 bpm and tachypnoeic with a respiratory rate of 28 and required intravenous fentanyl for pain management.

### 2.3. Laboratory Investigations

Initial laboratory workup revealed a profound systemic inflammatory response and secondary cardiac involvement. A marked leucocytosis of 23.2 × 10^9^/L was noted upon admission, which further increased to 26.3 × 10^9^/L the following day, characterised by a significant neutrophilia of 24.36 × 10^9^/L. Inflammatory markers were significantly elevated, C‐reactive protein (CRP) of 239 mg/L and erythrocyte sedimentation rate (ESR) of 84 mm/h. Cardiac biomarkers showed a troponin I elevation of 57 ng/L on admission, subsequently peaking at 156 ng/L during an episode of tachycardia. Although peripheral flow cytometry identified a small monoclonal CD8+ T‐cell expansion (0.4% of total cells), a comprehensive septic screen, including blood cultures and a respiratory viral panel, remained negative.

### 2.4. Radiological Imaging

A computed tomography pulmonary angiogram (CTPA) was performed to investigate the patient′s acute chest pain and elevated D‐dimer of 1.33 mg/L (Figure 1). Although negative for pulmonary embolism, the scan identified a massive, well‐circumscribed anterior mediastinal mass measuring 65 × 76 × 91 mm (Figure 1). The lesion demonstrated peripheral enhancement measuring 42HU surrounding a large hypoattenuating central component measuring 18HU, consistent with extensive internal necrosis. There was significant mass effect on cardiac structures, displacing the ascending aorta posteriorly and causing right ventricular (RV) dilatation with flattening of the interventricular septum.

**Figure 1:**
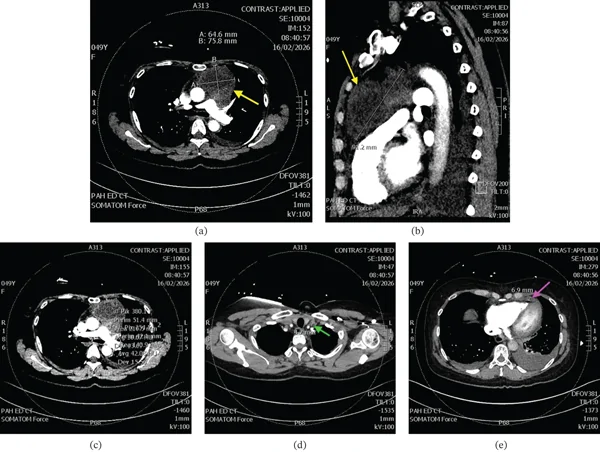
(a) Axial and (b) coronal reformatted CT images in the arterial phase demonstrate a large well‐circumscribed centrally hypoattenuating and peripherally enhancing mass (yellow arrows) within the anterior mediastinum measuring approximately 65 × 76 × 91 mm. (c) The lesion exhibits a peripheral enhancement measuring 42HU with large hypoattenuating central region (18HU) consistent with extensive internal necrosis. (d) Multiple prominent left supraclavicular lymph nodes (green arrow) measuring up to 5 mm in maximal dimension. (e) Small pericardial effusion (pink arrow) measuring up to 7 mm anteriorly was noted.

Further characterisation via transthoracic echocardiogram (TTE) confirmed extrinsic compression of the RV outflow tract and a 7 mm anterior pericardial effusion. A subsequent 18F‐FDG PET‐CT torso scan demonstrated moderate intense FDG avidity at the peripheral of the mass (SUV_max_ 7.27), whilst confirming the lack of metabolic activity in the central necrotic core (Figure 2). Crucially, the PET‐CT showed no evidence of pathologically enlarged or FDG‐avid lymphadenopathy above or below the diaphragm, effectively downstaging the suspicion for a metastatic primary or disseminated lymphoma.

**Figure 2:**
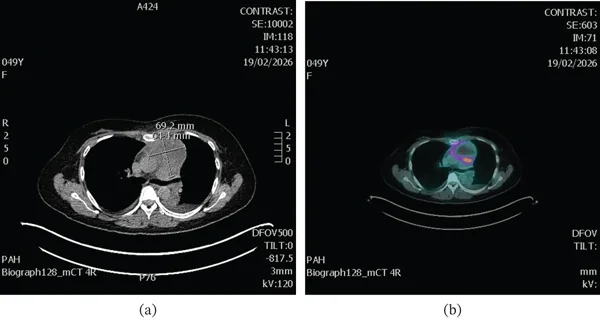
(a) Axial PET and (b) fused PET‐CT images demonstrate the metabolic profile of the anterior mediastinal mass. There is moderately intense tracer uptake localized to the peripheral rim of the lesion, with a maximum standardized uptake value (SUV_max_ of 7.27). The large central component is entirely photopenic, correlating with the necrotic/cystic changes seen on CT. No evidence of FDG‐avid lymphadenopathy or distant metastatic disease is identified above or below the diaphragm.

## 3. Pathological Analysis

The diagnostic workup began with a CT‐guided core biopsy obtaining six 18‐gauge cores. Histopathological slides revealed predominantly necrotic tissue with an “abscess‐like” quality, consisting of neutrophils, macrophages and granulation tissue. However, the pathologist noted an unusual sclerotic and compartmentalised background stroma, which was highly suspicious for a totally necrotic neoplasm. Immunohistochemistry for standard epithelial and lymphoid markers was largely non‐contributory.

Definitive diagnosis was achieved following surgical biopsy via a left‐sided Chamberlain procedure. Intraoperative findings revealed a thick‐walled, fluid‐filled lesion containing thick, black necrotic fluid. Large tissue fragments were harvested for formal analysis. Histopathology slides (Figure 3) comprise densely fibrotic tissue containing mixed chronic inflammation and areas of ectopic pancreatic tissue. The pancreatic tissue is arranged in normal‐appearing lobules and comprises acinar epithelium, islet cells and small ducts. Many of the small ducts appear dilated but overall, the tissue has a normal‐appearing architecture. All three constituents of the normal pancreas, acinar epithelium, ducts and islets of Langerhans, are represented, and the lesion is therefore classified as Heinrich Type I heterotopic pancreas [15]. In some pieces, there is admixed necrosis and haemorrhage. No other tissue types are included to suggest a teratoma. There is no evidence of lymphoproliferative disorder and no neoplasia.

**Figure 3:**
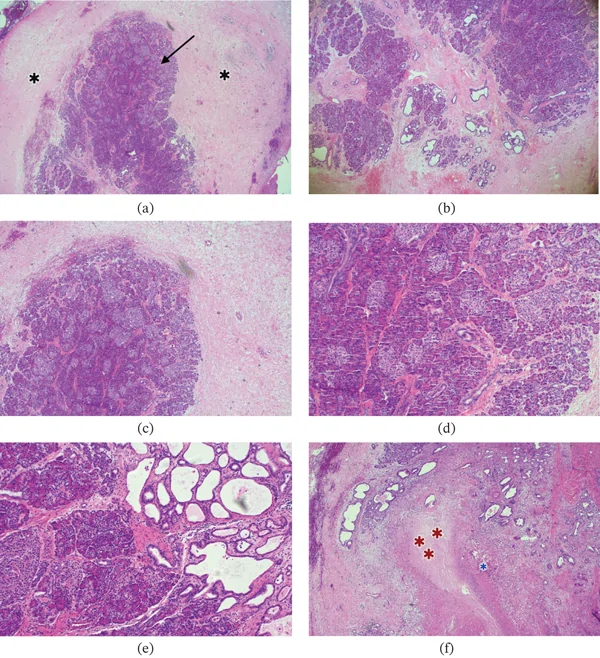
Histopathology of the ectopic pancreatic tissue in the mediastinum (haematoxylin and eosin staining throughout). (a) H&E, 20x and (b) H&E, 40x and (c) H&E, 40x: Wall of mediastinal mass comprises densely fibrotic tissue (∗) containing mixed chronic inflammation and areas of ectopic pancreatic tissue (black arrow). (d) H&E, 100x: The pancreatic tissue is arranged in normal‐appearing lobules and comprises acinar epithelium, islet cells, and small ducts. (e) H&E, 100x: Many of the small ducts appear dilated but overall, the tissue has a normal appearing architecture. (f) H&E, 100x: In some pieces there is admixed necrosis (red asterisks) and haemorrhage (blue asterisk).

Histopathological and radiological correlation of the surgical specimens ultimately confirmed the presence of organised pancreatic tissue within the mediastinum. The presence of the black, “ink‐like” fluid and extensive internal necrosis was consistent with enzymatic autodigestion, providing a clear radiological‐pathological correlation for this rare presentation of an ectopic mediastinal pancreas.

## 4. Treatment and Outcome

Following the pathologic confirmation of ectopic pancreatic tissue, the patient′s clinical course was complicated by difficulties with wound healing at the left‐sided anterior mini‐thoracotomy site from Chamberlain procedure related to pancreatic enzymatic action. She experienced erythema, pain, and persistent ooze from the surgical wound. Clinical assessment suggested that the discharge was consistent with fat necrosis caused by the ectopic pancreatic tissue oozing lytic enzymes, rather than a primary infection. She underwent urgent irrigation and debridement of the wound 7 days post Chamberlain procedure, which revealed a connection through the deeper suture layers into the mediastinum. A vacuum‐assisted closure dressing was subsequently applied.

Because of the persistent enzymatic activity and complications with the initial biopsy site, the decision was made to proceed with a definitive formal resection. Early April, the patient underwent a median sternotomy for the excision of the mediastinal mass. Intraoperatively, a moderate‐sized mass was identified in the mediastinum superior to the pulmonary artery. The mass was found to be densely adherent to the upper lobe of the left lung at the previous biopsy site, requiring a partial lung resection. The left phrenic nerve, brachiocephalic vein, and pericardium were carefully preserved during the dissection.

The patient′s postoperative recovery was excellent. By the fifth day following the sternotomy, her wounds were healing well, and she was mobilising independently. She was discharged with a plan for community suture removal and follow‐up review in the cardiothoracic clinic in 6 weeks. Notably, the cardiac arrhythmias that had characterised her initial presentation resolved following the removal of the inflammatory and mechanical triggers associated with the ectopic pancreatic tissue.

## 5. Discussion

The discovery of ectopic pancreatic tissue within the anterior mediastinum is an exceptionally rare clinical event, as this developmental anomaly is far more commonly situated within the distal foregut structures of the gastrointestinal tract [5–10]. In this case, the large 9.1 cm necrotic mass presented a significant diagnostic challenge, mimicking high‐grade malignancies such as thymoma or lymphoma, both clinically and radiologically.

### 5.1. Radiological–Pathological Correlation

The hallmark of this case was the correlation between the “thick, black necrotic fluid” encountered during the Chamberlain procedure and the specific Hounsfield unit observed on the initial CTPA. The central hypodensity of 18HU and peripheral hyperdensity of 42HU suggested a thick‐walled cystic structure rather than a solid tumour undergoing simple ischemic necrosis. Pathologically, ectopic pancreatic tissue remains functional and can undergo the same physiological and pathological processes as the native pancreas, including secretion of digestive enzymes. The extensive central necrosis and the presence of ink‐like black fluid are characteristic of enzymatic autodigestion occurring within the ectopic tissue. This enzymatic activity likely elicited the profound systemic inflammatory response—evidenced by the significantly elevated CRP and WCC, and the localised pericardial and pleural effusion observed on imaging.

### 5.2. Clinical Latency and Acute Presentation

A notable aspect of this case is why such a large mass remained clinically silent until the patient′s fifth decade. Although the lesion was likely congenital, arising from the primitive foregut, its recent symptomatic presentation was likely triggered by an acute inflammatory event or ectopic pancreatitis. This theory is supported by the patient’s history: she had no cardiac symptoms and a normal cardiomediastinal contour on CXR in December 2025, only to present 2 months later with a 9 cm mass and acute arrhythmias. The sudden expansion of the mass due to inflammation and enzymatic fluid accumulation explains the acute compression of the RV outflow tract. The mechanical pressure, combined with the systemic inflammatory state, served as the substrate for the new‐onset atrial fibrillation with rapid ventricular response and nonsustained ventricular tachycardia.

### 5.3. Tissue Sampling Considerations

The initial CT‐guided core biopsy returned non‐specific findings of abscess‐like necrotic tissue. The addition of amylase and lipase on subsequent sampling may have allowed the prompt diagnosis of ectopic pancreatic tissue.

### 5.4. Limitations

This report describes a single case and is retrospective in design. No intraoperative photographs were obtained at either procedure, and macroscopic documentation is therefore limited to the written operative and pathological records. Fluid from the lesion was not analysed for pancreatic enzymes, and formal quantification of the endocrine component of the ectopic tissue was not undertaken.

## 6. Conclusion

This case illustrates the radiologic and pathologic findings of rare large symptomatic anterior mediastinal ectopic pancreatic tissue mass. The sudden clinical manifestation highlights the potential for this congenital anomaly to undergo acute ectopic pancreatic necrosis and enzymatic autodigestion. For future undifferentiated mediastinal masses with similar features, clinicians should consider adding biochemical fluid analysis for pancreatic enzymes to expedite diagnosis.

## Funding

Open access publishing facilitated by The University of Queensland, as part of the Wiley–The University of Queensland agreement via the Council of Australasian University Librarians.

## Consent

Written consent has been obtained from the patient and has been reviewed by Queensland Health MSH Human Research Ethics Committee [HREC Reference Number: EX/2026/QMS/127867 (Mar ver 1)].

## Conflicts of Interest

The authors declare no conflicts of interest.

## Patient Perspective

The patient consented to publication of this report but did not provide a written personal statement.

## Supporting Information

Additional supporting information can be found online in the Supporting Information section.

## Supporting information


**Supporting Information 1** File S1: Queensland Health HREC approval letter.

**Supporting Information 2** File S2: Completed CARE checklist.

## Data Availability

The imaging and pathology slides that support the findings of this study are available on request from the corresponding author. The data are not publicly available due to privacy policy from Queensland Health and University of Queensland.
