# Supplementary material for: Case Report: Radiological–Pathological Correlation of Ectopic Pancreatic Tissue as Anterior Mediastinal Mass
Source: Case Rep Radiol. 2026 Sep 29;2026:4836145. doi: 10.1155/crra/4836145 (PMC13621400; doi:10.1155/crra/4836145)
Supplement: Supplementary file 1 [file CRRA-2026-4836145-s002.pdf]

Queensland Government

Metro South Health

Metro South Research

Enquiries to:	Metro South Research Ethics<br>MSH-Ethics@health.qld.gov.au
Telephone:	07 3443 8047 or 07 3443 8049
Our Ref:	EX/2026/QMS/127867 (Mar ver 1)
Date:	1 April 2026

To the attention of the project team,

**HREC Reference Number:**	**EX/2026/QMS/127867 (Mar ver 1)**
**Project Title:**	**Radiological-pathological correlation of ectopic pancreatic tissue as anterior mediastinal mass**

Thank you for submitting the above case report to the Metro South Health Human Research Ethics Committee (HREC) for ethical review. This application was considered by the Chair of the Metro South HREC and will be ratified at the next HREC meeting. Approval for publication has been granted.

Your project can proceed, subject to compliance throughout the duration of the project with the requirements as outlined in the *National Statement on Ethical Conduct in Human Research 2025* and the *Australian Code for the Responsible Conduct of Research (2018).*

I am sure you are aware of the importance of confidentiality issues and the need for any patient data to be non-identifiable in any publication and am satisfied that publication would be appropriate and is not in contravention of the NHMRC's National Statement.

A copy of this letter should be presented when required for publication purposes.

Should you have any queries about the Chair's consideration of your project please contact the Human Research Ethics Secretariat on 07 3443 8049.

Please note that the Metro South HREC is constituted and operates in accordance with the National Health and Medical Research Council's (NHMRC) *National Statement on Ethical Conduct in Human Research (2025), NHMRC and Universities Australia Australian Code for the Responsible Conduct of Research (2018)* and the *CPMP/ICH Note for Guidance on Good Clinical Practice*.

We wish you every success in undertaking this project.

Yours sincerely,

Mrs Sonia Hancock
HREC Chair
Metro South Health Human Research Ethics Committee (EC00167)
Metro South Research
Metro South Hospital and Health Service

Sites:

Princess Alexandra Hospital
