# Supplementary material for: Case Report: Radiological–Pathological Correlation of Ectopic Pancreatic Tissue as Anterior Mediastinal Mass
Source: Case Rep Radiol. 2026 Sep 29;2026:4836145. doi: 10.1155/crra/4836145 (PMC13621400; doi:10.1155/crra/4836145)
Supplement: Supplementary file 2 [file CRRA-2026-4836145-s001.docx]

# CARE Checklist — Completed

**Manuscript:** Case Report: Radiological–pathological Correlation of Ectopic Pancreatic Tissue as Anterior Mediastinal Mass

**Authors:** Quan Z, Luong D, Coucher J

**Journal:** Case Reports in Radiology · **Manuscript ID:** 4836145

**Checklist version:** CARE 2013 (Riley DS, Barber MS, Kienle GS, et al. CARE guidelines for case reports: explanation and elaboration document. *J Clin Epidemiol.* 2017;89:218–35)

| # | Topic | Checklist item | Reported | Location in manuscript |
| --- | --- | --- | --- | --- |
| 1 | **Title** | The diagnosis or intervention of primary focus followed by the words “case report” | Yes | Title page. “Case Report: Radiological–pathological Correlation of Ectopic Pancreatic Tissue as Anterior Mediastinal Mass” |
| 2 | **Key Words** | 2 to 5 key words that identify diagnoses or interventions in this case report, including “case report” | Yes | Following the Abstract. Five keywords: case report; ectopic pancreas; heterotopic pancreas; anterior mediastinal mass; radiological–pathological correlation |
| 3a | **Abstract** | Introduction — what is unique about this case and what it adds to the literature | Yes | Abstract, sentence 1 |
| 3b | **Abstract** | Main symptoms and/or important clinical findings | Yes | Abstract, sentences 2–4 |
| 3c | **Abstract** | Main diagnoses, therapeutic interventions and outcomes | Yes | Abstract, sentences 5–6 |
| 3d | **Abstract** | Conclusion — what are the main “take-away” lessons | Yes | Abstract, final sentence |
| 4 | **Introduction** | Brief background summary of this case referencing the relevant medical literature | Yes | Introduction, full section. Fifteen references cited |
| 5a | **Patient Information** | De-identified demographic and other patient-specific information | Yes | Case Presentation – Initial Presentation. 49-year-old female; no identifying detail reported; dates reduced to month and year |
| 5b | **Patient Information** | Primary concerns and symptoms of the patient | Yes | Case Presentation – Initial Presentation and Acute Representation; Table 1 |
| 5c | **Patient Information** | Medical, family and psychosocial history including relevant genetic information | Partial* | Case Presentation – Initial Presentation. No relevant family or genetic history was identified; ectopic pancreas is a sporadic congenital anomaly without recognised heritable predisposition |
| 5d | **Patient Information** | Relevant past interventions and their outcomes | Yes | Case Presentation – Initial Presentation. No relevant prior interventions; the December 2025 attendance concluded with outpatient investigation only |
| 6 | **Clinical Findings** | Describe significant physical examination and important clinical findings | Yes | Case Presentation – Initial Presentation (normal neurological and cranial nerve examination) and Acute Representation (vital signs, rhythm) |
| 7 | **Timeline** | Historical and current information from this episode of care organised as a timeline (figure or table) | Yes | **Table 1**, cited at the opening of the Case Presentation |
| 8a | **Diagnostic Assessment** | Diagnostic testing, including physical examination, laboratory testing and imaging | Yes | Case Presentation – Laboratory Investigations, Radiological Imaging, Pathological Analysis; Figures 1–3; Table 1 |
| 8b | **Diagnostic Assessment** | Diagnostic challenges — access, financial, cultural | Yes | Case Presentation – Pathological Analysis (non-diagnostic core biopsy); Discussion – Tissue sampling considerations |
| 8c | **Diagnostic Assessment** | Diagnostic reasoning including other diagnoses considered | Yes | Introduction (thymoma, germ cell tumour, lymphoma); Case Presentation – Radiological Imaging and Pathological Analysis (exclusion of teratoma and lymphoproliferative disorder); Discussion |
| 8d | **Diagnostic Assessment** | Prognostic characteristics where applicable | Yes | Discussion – Clinical latency and acute presentation; Treatment and Outcome |
| 9a | **Therapeutic Intervention** | Types of intervention — pharmacologic, surgical, preventive, self-care | Yes | Case Presentation – Treatment and Outcome. CT-guided core biopsy; Chamberlain procedure; wound irrigation and debridement with vacuum-assisted closure; median sternotomy with excision and partial lung resection |
| 9b | **Therapeutic Intervention** | Administration of intervention — dosage, strength, duration | Yes | Case Presentation – Acute Representation and Treatment and Outcome. Surgical interventions described with approach, timing and extent |
| 9c | **Therapeutic Intervention** | Changes in intervention with rationale | Yes | Case Presentation – Treatment and Outcome. Escalation from percutaneous biopsy to Chamberlain procedure, and from biopsy to definitive resection, each with stated rationale |
| 10a | **Follow-up and Outcomes** | Clinician- and patient-assessed outcomes where appropriate | Yes | Case Presentation – Treatment and Outcome; Table 1, final row |
| 10b | **Follow-up and Outcomes** | Important follow-up diagnostic and other test results | Yes | Case Presentation – Treatment and Outcome. Resolution of arrhythmia following resection |
| 10c | **Follow-up and Outcomes** | Intervention adherence and tolerability, and how this was assessed | Yes | Case Presentation – Treatment and Outcome. Postoperative course and mobilisation described |
| 10d | **Follow-up and Outcomes** | Adverse and unanticipated events | Yes | Case Presentation – Treatment and Outcome. Wound dehiscence and enzymatic fat necrosis at the mini-thoracotomy site, with management described |
| 11a | **Discussion** | Strengths and limitations in the management of this case | Yes | Discussion – Limitations |
| 11b | **Discussion** | Discussion of the relevant medical literature | Yes | Discussion, full section |
| 11c | **Discussion** | Rationale for conclusions, including assessment of possible causes | Yes | Discussion – Radiological–pathological correlation; Clinical latency and acute presentation |
| 11d | **Discussion** | Primary “take-away” lessons from this case report | Yes | Conclusion |
| 12 | **Patient Perspective** | The patient should share their perspective on the treatment(s) they received | Yes* | Not obtained; stated in the patient perspective section after consent declaration |
| 13 | **Informed Consent** | The patient should give informed consent (provide if requested) | Yes | Consent section. Written consent obtained and reviewed by the Queensland Health Metro South Health Human Research Ethics Committee, HREC reference EX/2026/QMS/127867 (Mar ver 1). Available on request |

## Notes

**Item 5c (medical, family and psychosocial history).** Marked partial. Ectopic pancreas arises from a sporadic error of embryological migration and has no recognised heritable basis, so a family history is not informative in the way it would be for a neoplastic diagnosis. A single explanatory sentence can be added to the Case Presentation if the Editor prefers.

**Item 12 (patient perspective).** The patient consented to publication of this report but did not provide a written personal statement.

## Declaration

The authors confirm that this case report has been prepared and reported in accordance with the CARE guidelines, and that this checklist accurately reflects the content of the submitted manuscript.

**Corresponding author:** Zehao Quan · Zehao.quan@student.uq.edu.au

**Date:** 13 Aug 2026
